# The relationship between spasticity in young children (18 months of age) with cerebral palsy and their gross motor function development

**DOI:** 10.1186/1471-2474-10-108

**Published:** 2009-09-04

**Authors:** Jan Willem Gorter, Olaf Verschuren, Laura van Riel, Marjolijn Ketelaar

**Affiliations:** 1 *Canchild*, Centre for Childhood Disability Research, Institute for Applied Health Sciences, McMaster University, Hamilton, Ontario, Canada; 2 Partner of NetChild, Network for Childhood Disability Research, The Netherlands; 3 Centre of Excellence, Rehabilitation Centre 'De Hoogstraat', Utrecht, The Netherlands; 4 Rudolf Magnus Institute of Neuroscience, Department of Rehabilitation and Sports Medicine, University Medical Centre Utrecht, Utrecht, The Netherlands

## Abstract

**Background:**

It is thought that spasticity has an influence on the development of functional motor abilities among children with cerebral palsy (CP). The extent to which spasticity is associated with the change in motor abilities in young children with CP has not been established. The objective of this study is to evaluate the relationship of initial spasticity in young children with CP and their gross motor function development over one year.

**Methods:**

Fifty children with CP aged 18 months, GMFCS-levels I-V participated in a longitudinal observational study. Change in gross motor functioning (GMFM-66) was measured over one year. The level of spasticity measured at the first assessment was determined with the Modified Tardieu Scale in three muscle groups of the lower extremity (adductor muscles, the hamstrings and the m. gastrocnemius). The Spasticity Total Score per child was calculated with a maximum score of 12 points.

**Results:**

Spearman's Rho Correlation (-0.28) revealed a statistically significant relationship (p < 0.05) of small strength between the Spasticity Total Score and the change score of the GMFM-66.

**Conclusion:**

Our findings indicate that when measured over one year, spasticity is marginally related to gross motor function development in infants with CP. The initial level of spasticity is only one of the many child, environmental and family factors that determines gross motor development of a young child with CP.

## Background

Cerebral palsy (CP) describes a group of permanent disorders, effecting the development of movement and posture, causing activity limitation, that are attributed to non-progressive disturbances that occurred in the developing fetal or infant brain [1]. The motor disorders of CP are often accompanied by disturbances in sensation, perception, cognition, communication, and behaviour, as well as epilepsy and secondary musculoskeletal problems [1].

Motor impairment in CP can be classified into three different subtypes, i.e. spastic, dyskinetic or the ataxic form [2]. The most common movement disorder in CP is spastic paresis, defined as a posture and movement dependent tone regulation disorder [3]. A broad diversity of muscle function impairments can be present in spastic paresis. Clinical symptoms of impaired muscle function can either be related to an impairment of muscle activation, leading to both deficit (or negative) symptoms (e.g. paresis, loss of voluntary selective motor control) and excess (or positive) symptoms (e.g. spasticity), or to a change in biomechanical properties of muscles and connective tissues [4]. The most prominent symptom in spastic paresis is spasticity. According to a generally accepted and quoted definition of spasticity in both adult and paediatric literature, spasticity is defined as 'a motor disorder characterized by a velocity dependent increase in tonic stretch reflexes (muscle tone) with exaggerated tendon jerks, resulting from hyper-excitability of the stretch reflex, as one component of the upper motor neuron syndrome' [5,6]. Others, e.g. members of the SPASM consortium, have tried to redefine spasticity to encompass the current understanding of pathophysiology and clinical practice [6]. There is a need for evidence that this wider definition is useful and that measures of spasticity are congruent with the definition [6]. In clinical practice, spasticity is assessed as a velocity-dependent increased resistance to passive muscle stretch.

Despite limited knowledge about the relationship between spasticity and motor abilities, many intervention strategies focus on reduction of spasticity, with the assumption that reduction of spasticity will lead to an increase in motor abilities. There is limited evidence however, that reduction of spasticity in children with CP is related to a better development [7]. Therefore, knowledge of the nature and strength of relationships between the initial impairments in movement, posture and (change in) performance in a child with CP is very important to serve as a base for rehabilitation interventions. Recent cross-sectional studies in children with CP have shown a modest, negative relationship between spasticity and daily activities in children with CP aged three years and older [8-10]. Also in one botulinum toxin type A (BoNT-A) intervention study in 35 children (mean age 5 years) it was found that at baseline spasticity was poorly related to the 66-item version of the Gross Motor Function Measure (GMFM-66) for the gastrocnemius muscles (r < 0.35) and moderately related to the GMFM-66 for the hamstrings (r = -0.55). Moreover, change score correlations between spasticity and GMFM-66-score at each of 2 months and 6 months was no more than fair (i.e. maximum r < 0.40) [11]. In conclusion, spasticity - as a primary impairment in CP - is thought to have an influence on the development of the functional motor abilities, but the extent to which spasticity is related with the change in motor abilities in infants with CP has not been established in a study with a longitudinal design.

The aim of this study is to evaluate the relationship between initial spasticity in the lower limbs of young children (under the age of two years) with CP and the development of their gross motor function over one year. Based on the evidence in the literature we hypothesize that first, spasticity early in life has a negative relationship with gross motor development. Second, the strength of the correlation between initial spasticity and gross motor development is modest, accounting for no more than 16% of explained variation in the change score of the 66-item version of the Gross Motor Function Measure (GMFM-66).

## Methods

The present study is part of the PERRIN CP 0-5 study, which, in turn, is part of a longitudinal research program entitled Pediatric Rehabilitation Research in the Netherlands (PERRIN). In the PERRIN program, the course and determinants of the functional status of children and adolescents with CP are studied . The focus in PERRIN CP 0-5 is on the development of activities and participation of young children with CP starting at age 18 months. The protocol has been approved by the Ethics Committee of the University Medical Center Utrecht and by other participating rehabilitation centres and hospitals in the Netherlands (see acknowledgements).

Parents were asked by the paediatrician, child neurologist or rehabilitation physician of their child whether they were willing to participate in the PERRIN CP 0-5 study.

Between April 2003 and December 2006, 77 children under two years of age, corrected age if applicable, were eligible for the present study. Written consent was obtained from 63 mothers and fathers (82% response rate). The diagnosis of CP was evaluated and, in the case of uncertainty checked by an independent neurologist. Children who had additional disorders (besides CP) which had an important and persistent influence on the motor abilities (e.g. Spina Bifida, cardiac impairments, severe bronchopulmonal dysplasia) and parents with insufficient knowledge of the Dutch language which could hamper assessments, were excluded from the study. For the current study, complete data on the GMFM and spasticity were available in 50 out of 63 children at 18 months of age participating in the PERRIN

CP 0-5 study. The group of participants consisted of children with a variety in type of the predominant motor disorder, the numbers of limbs involved (uni- versus bilateral involvement according to SCPE guidelines) [2] and the level of gross motor abilities, classified according to the GMFCS (Gross Motor Function Classification System) [12]. For this study we used the GMFCS descriptions provided for children aged 2 to 4 years at time 2 as being a more precise classification than the classification for infants under 2 years of age [13]. GMFCS Level I represents the highest level of functional abilities and GMFCS Level V the lowest. The baseline characteristics of participants are presented in Table 1.

**Table 1:** Baseline characteristics

	**Frequency (%)** **N = 50**
**Gender**	

Boy	27 (54)

Girl	23 (46)

**Classification CP**	

Spastic bilateral	27 (54)

Spastic unilateral right	11 (22)

Spastic unilateral left	11 (22)

Dyskinetic	1 (2)

**GMFCS level**	

I	14 (28)

II	7 (14)

III	13 (26)

IV	10 (20)

V	6 (12)

### Procedure

Gross motor function was assessed at the age of 18 months (t1) and one year later (t2). Initial spasticity was assessed at t1. All assessments were performed according to the study protocol by a team of trained research assistants with a background in physiotherapy, movement sciences or medicine. To monitor the reliability of the GMFM, all of the assessors were tested using a criterion test videotape.

### Instruments

#### Gross motor function

Gross motor function of all children was assessed using the Gross Motor Function Measure-66 (GMFM-66) [14]. The GMFM-88 is a standard criterion-referenced measure for detecting and monitoring changes in motor functions [15]. The 88 items of the test assess activities in five dimensions. Each item is scored using a 4-point Likert scale (0-3 with 0 representing the lowest performance level and 3 the highest). The GMFM-66 uses 66 of the 88 items and was developed using Rasch analysis to improve the sensitivity and interpretability of the test [14]. For each child the values of the GMFM-88 were analyzed using the Gross Motor Ability Estimator computing scoring program to acquire an interval-level GMFM-66 score ranging from 0 to 100 (maximum score) which represents a child's overall level of gross motor functions [14,15]. The GMFM-66 is a valid and reliable measure [15] with responsiveness to change [16].

To evaluate the development of gross motor function in one year, the interval-level GMFM-66 scores of the first measurement moment (t1) were deducted from the GMFM-66 scores of the second measurement moment (t2). The result of this deduction was called the GMFM-66 Change Score and reflects the change in GMFM-66 score in one year (i.e. the progression or regression of gross motor functioning in one year).

#### Spasticity

Spasticity of the muscle groups that are most relevant for gross motor function of the lower extremities (adductor muscles, the hamstrings and the m. gastrocnemius) were assessed with the Modified Tardieu Scale (MTS) [17], which is a modification of the Tardieu scale [18]. The MTS grades spasticity by measuring the joint angle of the ROM at which an increase in muscle tone ('catch') is encountered at a high velocity (< 1 sec) passive stretch (R1). Acceptable inter-rater reliability of the MTS has been reported in two studies in children with CP [11,19]. In a third study, however, the intra-class correlation coefficient of the MTS in children with spastic CP did not reach the acceptable limit of good reliability [20].

Because the impact on motor function of spasticity is not the same for the different muscle groups that were assessed, a rough ordinal scale (with 3 different levels) of spasticity was created. This scale was created on the opinion of five experts in the field of spasticity in children with CP (a rehabilitation physician, two physical therapists and two rehabilitation researchers) and the available information about the variance in assessing spasticity with the MTS [19]. The first level of the spasticity scale (no spasticity, level 0) was determined by the measurement error per muscle group in the literature, our own data and expert opinion. Fosang et al. found an inter-rater variance of approximately 10 degrees in measuring the adductor muscles at a high velocity passive stretch, 20 degrees in measuring the hamstrings and 15 degrees in measuring the gastrocnemius muscle in children with CP (age range 2 years 4 months to 10 years)[19] The inter-rater difference was calculated from data gathered in order to determine the 'measurement error' in the study's measurements. This measurement error corresponded with the measurement error that was the result of the expert opinion round. The expert panel considered a measurement error of 0-10° for the adductors and gastrocnemius and 0-20° for the hamstrings as an acceptable clinical cut-off point. To specify the second level of spasticity (probable spasticity, level 1) in a spasticity scale, the collected data of the three muscle groups (left and right) of the included participants were statistically analyzed for mean, range and standard deviation. Of each separate muscle group 1 standard deviation (SD) corresponded with the 'middle' range of spasticity as indicated by the expert-panel, and values more than 1 SD corresponded with the definite presence of spasticity (level 2). In summary, based on the aforementioned procedure an ordinal spasticity scale with three levels of spasticity was developed (Table 2).

**Table 2:** Classification of spasticity

**Level of spasticity**	**Score**	**Cut-off points for R1 (in degrees per muscle group)**	**Range(°)**
***ADDUCTORS***			

No spasticity	0	0-10	

Probable spasticity	1	11-20	1 SD (Right:10.0 Left:10.1)

Definite spasticity	2	21 >	

**HAMSTRINGS**			

No spasticity	0	0-20	

Probable spasticity	1	21-40	1 SD (Right: 17.7, Left: 18.0)

Definite spasticity	2	41 >	

**GASTROCNEMIUS**			

No spasticity	0	0-10	

Probable spasticity	1	11-25	1 SD (Right 13.4, Left 13.0)

Definite spasticity	2	26 >	

Classification with 3 different levels of spasticity per muscle group based on the measurement error per muscle group in the literature (Fosang et al. [19]) and expert opinion.

Furthermore, the spasticity-scale was slightly adjusted based on the group of participants, who are children with CP in the age 1.5 years (Table 3). For example the adductor muscles: according to Fosang et al[19], the measurement error was 0-10 degrees. The standard full range of motion of the adductor muscles is 70 degrees. However, in this study participant's largest abduction angle (ROM of the adductor muscles) measured was 90 degrees. Therefore, the range of error of measurement ('level 0') was adjusted to a range of 90-60 degrees. The middle range of spasticity in the adductors was 10 degrees (1SD). The middle range ('level 1') of the classification was 60-51 degrees, and 'level 2' was valued as 50 degrees or less.

**Table 3:** Classification of spasticity applied to the obtained data in this study.

**Level of spasticity**	**Score**	**Cut-off points for the angle of spasticity (R1) in our group (in degrees per muscle group)**	**Range**
**ADDUCTORS**		**Abduction**	

No spasticity	0	61.>	

Probable spasticity	1	60-51	1 SD (Right:10.0 Left:10.1)

Definite spasticity	2	≤ 50	

**HAMSTRINGS**		**Popliteal angle**	

No spasticity	0	0-20	

Probable spasticity	1	21-40	1 SD (Right: 17.7, Left: 18.0)

Definite spasticity	2	≥ 41	

**GASTROCNEMIUS**		**Ankle-foot position**	

No spasticity	0	11 - 50 dorsiflexion	

Probable spasticity	1	10 dorsiflexion - (-5)plantarflexion	1 SD (Right 13.4, Left: 13.0)

Definite spasticity	2	≤ (-5) plantarflexion	

Data based on spasticity (R1 angle) per muscle group from young children with cerebral palsy.

Finally, the angles of spasticity in degrees (R1) were classified according to these three levels. Adding these levels for all muscle groups (right and left side) resulted in a maximal score of 12 points. The total value of spasticity per child was named Spasticity Total Score.

### Statistical analysis

Statistical analysis of data was carried out using SPSS-16 (SPSS, Chicago, Illinois, USA). The relationship between the Spasticity Total Score and the GMFM-66 Change Score was expressed by Spearman's Rho. The data were analyzed for the total group (n = 50). We refrained from undertaking additional subgroup analysis because of the difficulty to classify the type of motor disorder and its distribution in children with CP under the age of 2 years accurately. For interpreting the values of the strength of the correlation the guidelines of Cohen [21] were used. A Spearman's Rho of 0.10 to 0.29 counts as a small, a Rho of 0.30 to 0.49 as a medium and a Rho of 0.50 to 1.00 as a large correlation. The Spearman's Rho square indicates how much variation of the GMFM-66 Change Score was explained by the ordinal Spasticity Total Score.

## Results

According to the Spasticity Total Score we used, 17 of the 50 children did not have spasticity at their first assessment. One of these 17 children was classified as dominant dyskinetic movement disorder, five as spastic CP with bilateral involvement and 11 as spastic CP with unilateral involvement.

**Figure 1:**
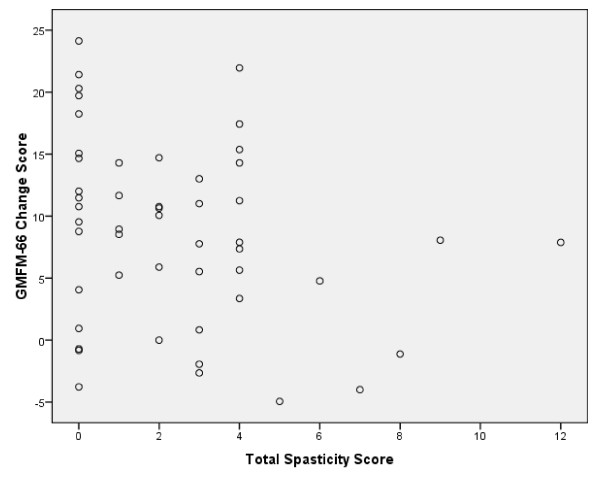
**Observed relationship between spasticity and gross motor function development**. Initial Spasticity Total Score (minimal score: 0, maximum score: 12) per child (age 18 months) and the change in GMFM 66 score in one year (n = 50). Spearman's Rho Correlation is -0.28 (p = 0.05); Square Spearman's Rho = 0.08. Approximately 8% of the GMFM change score is explained by the Spasticity Total Score.

The relationship between initial spasticity (Spasticity Total Score) and the GMFM-66 development in one year (GMFM-66 Change Score) is reflected in Figure 1. Spearman's Rho correlation (-0.28) revealed a statistically significant (p < 0.05) relationship of small strength between the assessed total spasticity and the Change Score of the GMFM 66. The square Spearman's Rho was 0.08.

## Discussion

In the group of children with CP included in this study, the data gathered indicates that there is a statistically significant relationship between spasticity at the age 18 months and gross motor development over one year. Only 8% of the variance in change in motor function could be explained by spasticity as primary impairment. As shown in Figure 1, the change in motor score in the absence of spasticity (total spasticity score = 0) could vary between a 24 point increase (a child with unilateral spastic CP, GMFCS level I) to a decrease of almost 4 points (a child with bilateral spastic CP, GMFCS level V), while a child with severe spasticity in all three muscle groups (total spasticity score = 12, GMFCS level III) had an increase of 8 points in GMFM-score in one year. Both of our hypothesis in this study were confirmed. This study reveals that spasticity early in life is a minimal factor in functional change when considered alone and would be probably less when other child, environment and family factors are included.

In general, assessment of spasticity is difficult. Valid and reliable assessment of spasticity in young children is even more a challenge, and the most common spasticity scales should be interpreted with caution [22,23]. The Ashworth scale (AS), Modified Ashworth Scale (MAS), Tardieu scale and Modified Tardieu Scale are the most reported measures in children with CP. According to the review by Scholtes et al. [23] the original Tardieu Scale is the only suitable instrument to measure spasticity because it measures a velocity dependent resistance at three specified velocities. This method is very time consuming and would interfere with the feasibility of using this instrument in young children.

A limitation of the current study is that it was not possible to study the inter-rater reliability of the Modified Tardieu Scale as the assessment was part of a larger comprehensive assessment in young children within the PERRIN CP 0-5 study. Moreover, the assessments have been performed by a team of investigators with different backgrounds and levels of experience, which may result in a higher variance in measurements than in the study of Kilgour et al. [24]. When assessing spasticity with the Modified Tardieu Scale, Fosang et al. [19] found inter-rater differences of approximately 10 degrees for the adductors, 20 degrees for the hamstrings and 10-15 degrees for the gastrocnemius muscle. These reliability findings are similar with our assessors. Furthermore, with the measurement error taken into account by the recoding of the obtained R1 angles for each muscle group to the spasticity score, it was felt that inter-rater measurement errors - if any - were taken into account for this population. For the study our own composite spasticity score based on the MTS was developed. As a consequence, we do not have data on the reliability of the Spasticity Total Score in young children with CP. A few studies have been published in which classification of spasticity with the Modified Ashworth Scale has been given in adult stroke patients [25,26]. By adjusting the MAS to a 0-5 scale and applying this scale to the affected flexors at the elbow, wrists and fingers a "Composite Spasticity Index' from 0-15 was made. Ibrahim and Hawamdeh [27] used the same 'Composite Spasticity Index' as created by Francis et al. [25] in a group of children with CP. The 0-5 scale was applied to the spastic hip adductors and the spastic knee extensors. Despite its frequent use in clinical practice, the validity and the reliability of the MAS as a measure of spasticity, however, is questioned [22].

The results of this study are in line with other studies on the modest relationship between spasticity (ICF body function level) and capabilities/performance (ICF activities and participation level) in other pediatric populations[8-11] The strengths of this study are the young age of children with CP (starting under two years of age), a valid instrument with acceptable inter-rater reliability for assessing spasticity (MTS) and a valid and reliable instrument for assessing change in gross motor abilities (GMGM-66). All data collected are from a large clinical based sample of young children diagnosed as having CP, who were receiving a range of accepted medical physical therapy and rehabilitation services. It is believed the sample is representative of the population of young children with CP in The Netherlands, with results able to be generalized to populations in the same age group elsewhere receiving similar types of mixed developmental therapies.

**Figure 2:**
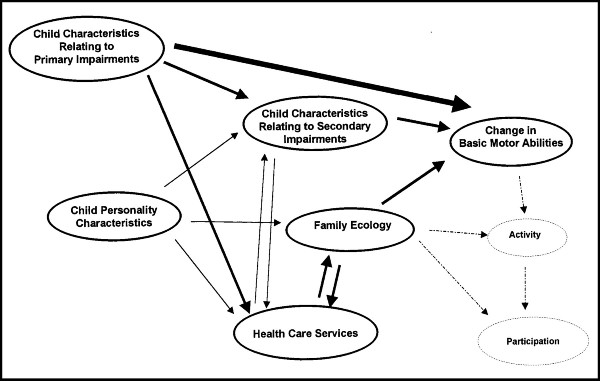
**Model of determinants of motor change for children with cerebral palsy**. Solid lines and ovals are part of the proposed model; dotted lines and ovals represent parts of an expanded model as proposed by Bartlett and Palisano.^11 ^(With permission of Physical Therapy).

The small square Spearman's Rho for all CP subtypes combined indicates that there seems to be more variables determining the development of the gross motor function over one year than spasticity alone. The overall relationship between spasticity and gross motor development may have been overestimated since 22 children with unilateral spastic CP and their data from both lower extremities were included in the analysis. However, the Spasticity Total Score in children with unilateral CP ranged from 0 to 4 indicating that the presumed unaffected side is really not unaffected in these children. The association between spasticity and gross motor function would probably be even less when the analysis included other possible child characteristics such as deficit symptoms (muscle weakness [10], loss of voluntary selective motor control), disturbances of sensation, perception, and cognition. Furthermore, if secondary musculoskeletal impairments occur, these factors also can have an influence on the development of gross motor function. Bartlett and Palisano introduced a multivariate model of determinants of motor change for children with CP, based on literature and expert opinion (Figure 2) [28]. In this model possible determinants of motor change include secondary impairments, personal and contextual factors (family ecology) and interventions. Since the presence and severity of contractures are limited in children aged 1 year of age, it is felt that secondary impairments are of minor influence on the results.

The results do not imply that reducing spasticity in a child with CP can not be beneficial in the attainment of gross motor skills or in the prevention of secondary impairments that could negatively influence the level of motor abilities. Spasticity treatment with botulinum toxin in combination with intensive physical therapy has been shown to be effective, not only in reducing spasticity in the short term, but also in promoting improvement in motor abilities as measured with the GMFM in both young and older children [29]. The study looked at the relationship between initial spasticity and gross motor development occurring over one year in young children with CP under two years of age. A recent study indicates that in children with CP the muscle tone in the gastrocnemius muscle increases significantly from 1 to 4 years of age [30]. To look at the role of spasticity and its relationship with the development of gross motor function and contractures in the long-term these children should be followed longitudinally for a longer period of time.

## Conclusion

In conclusion, the findings indicate that spasticity is marginally related to the development of gross motor function in one year in children with CP under the age of two. Knowledge about determinants of gross motor performance may be useful to set realistic rehabilitation goals and provide both patients and parents complete and reliable information about the expected effects of spasticity treatment.

## Competing interests

The authors declare that they have no competing interests.

## Authors' contributions

JWG and OV designed the study. MK participated in the data-collection and statistical analysis. OV and LvR analyzed the results. OV wrote the first draft of the manuscript, which was reviewed by all authors. JWG revised the manuscript. All authors read and approved the final version.

## Authors' information

JWG is Principal Investigator of the PERRIN CP 0-5 study. MK is co-ordinator of the PERRIN research program.

## Pre-publication history

The pre-publication history for this paper can be accessed here:



## References

[B1] Rosenbaum P, Paneth N, Leviton A, Goldstein M, Bax M, Damiano D (2007). A report: The definition and classification of cerebral palsy April 2006. Dev Med Child Neurol Suppl.

[B2] Surveillance of Cerebral Palsy in Europe (SCPE) (2000). Surveillance of Cerebral Palsy in Europe: a collaboration of cerebral palsy surveys and registers. Dev Med Child Neurol.

[B3] Hagberg B, Hagberg G, Beckung E, Uvebrant P (2001). Changing panorama of cerebral palsy in sweden. VIII. prevalence and origin in the birth year period 1991-94. Acta Paediatr.

[B4] Becher JG, Harlaar J, Lankhorst GJ, Vogelaar TW (1998). Measurement of impaired muscle function of the gastrocnemius, soleus, and tibialis anterior muscles in spastic hemiplegia: A preliminary study. J Rehabil Res Dev.

[B5] Lance JW (1980). Symposium synopsis. spasticity: Disordered motor control.

[B6] Malhotra S, Pandyan A, Day C, Jones P, Hermens H (2009). Spasticity, an impairment that is poorly defined and poorly measured. Clin Rehabil.

[B7] Butler C, Darrah J (2001). Effects of neurodevelopmental treatment (NDT) for cerebral palsy: An AACPDM evidence report. Dev Med Child Neurol.

[B8] Ostensjo S, Carlberg EB, Vollestad NK (2003). Everyday functioning in young children with cerebral palsy: Functional skills, caregiver assistance, and modifications of the environment. Dev Med Child Neurol.

[B9] Abel MF, Damiano DL, Blanco JS, Conaway M, Miller F, Dabney K (2003). Relationships among musculoskeletal impairments and functional health status in ambulatory cerebral palsy. J Pediatr Orthop.

[B10] Ross SA, Engsberg JR (2007). Relationships between spasticity, strength, gait, and the GMFM-66 in persons with spastic diplegia cerebral palsy. Arch Phys Med Rehabil.

[B11] Wright FV, Rosenbaum PL, Goldsmith CH, Law M, Fehlings DL (2008). How do changes in body functions and structures, activity, and participation relate in children with cerebral palsy?. Dev Med Child Neurol.

[B12] Palisano R, Rosenbaum P, Walter S, Russell D, Wood E, Galuppi B (1997). Development and reliability of a system to classify gross motor function in children with cerebral palsy. Dev Med Child Neurol.

[B13] Gorter JW, Ketelaar M, Rosenbaum P, Helders PJ, Palisano R (2009). Use of the GMFCS in infants with CP: the need for reclassification at age 2 years or older. Dev Med Child Neurol.

[B14] Russell DJ, Avery LM, Rosenbaum PL, Raina PS, Walter SD, Palisano RJ (2000). Improved scaling of the gross motor function measure for children with cerebral palsy: Evidence of reliability and validity. Phys Ther.

[B15] Russell DJ, Rosenbaum PL, Avery LM, Lane M (2002). Gross motor function measure (GMFM-66 & GMFM-88) User's manual.

[B16] Vos-Vromans DC, Ketelaar M, Gorter JW (2005). Responsiveness of evaluative measures for children with cerebral palsy: The gross motor function measure and the pediatric evaluation of disability inventory. Disabil Rehabil.

[B17] Boyd RN, Graham HK (1999). Objective measurement of clinical findings in the use of botulinum toxin type a for the management of children with cerebral palsy. Eur J Neurol.

[B18] Held JP, Pierrot-Deseilligny E (1969). Le bilan moteur central.

[B19] Fosang AL, Galea MP, McCoy AT, Reddihough DS, Story I (2003). Measures of muscle and joint performance in the lower limb of children with cerebral palsy. Dev Med Child Neurol.

[B20] Yam WK, Leung MS (2006). Interrater reliability of modified ashworth scale and modified tardieu scale in children with spastic cerebral palsy. J Child Neurol.

[B21] Cohen J (1988). Statistical power analysis for the behavioral sciences.

[B22] Mutlu A, Livanelioglu A, Gunel MK (2008). Reliability of ashworth and modified ashworth scales in children with spastic cerebral palsy. BMC Musculoskelet Disord.

[B23] Scholtes VA, Becher JG, Beelen A, Lankhorst GJ (2006). Clinical assessment of spasticity in children with cerebral palsy: A critical review of available instruments. Dev Med Child Neurol.

[B24] Kilgour G, McNair P, Stott NS (2003). Intrarater reliability of lower limb sagittal range-of-motion measures in children with spastic diplegia. Dev Med Child Neurol.

[B25] Francis HP, Wade DT, Turner-Stokes L, Kingswell RS, Dott CS, Coxon EA (2004). Does reducing spasticity translate into functional benefit? an exploratory meta-analysis. J Neurol Neurosurg Psychiatry.

[B26] Bohannon RW, Smith MB (1987). Interrater reliability of a modified ashworth scale of muscle spasticity. Phys Ther.

[B27] Ibrahim AI, Hawamdeh ZM (2007). Evaluation of physical growth in cerebral palsied children and its possible relationship with gross motor development. Int J Rehabil Res.

[B28] Bartlett DJ, Palisano RJ (2000). A Multivariate Model of Determinants of Motor Change for Children With Cerebral Palsy. Phys Ther.

[B29] Scholtes VA, Dallmeijer AJ, Knol DL, Speth LA, Maathuis CG, Jongerius PH (2007). Effect of multilevel botulinum toxin a and comprehensive rehabilitation on gait in cerebral palsy. Pediatr Neurol.

[B30] Hagglund G, Wagner P (2008). Development of spasticity with age in a total population of children with cerebral palsy. BMC Musculoskelet Disord.

